# Genomic Prediction for Winter Survival of Lowland Switchgrass in the Northern USA

**DOI:** 10.1534/g3.119.400094

**Published:** 2019-04-10

**Authors:** Hari P. Poudel, Millicent D. Sanciangco, Shawn M. Kaeppler, C. Robin Buell, Michael D. Casler

**Affiliations:** * Department of Agronomy, University of Wisconsin-Madison, Madison, WI 53706; † Great Lakes Bioenergy Research Center, Michigan State University, East Lansing, MI 48824; ‡ Department of Plant Biology, Plant Resilience Institute, and MSU AgBioResearch, Michigan State University, East Lansing, MI 48824; § United States Dairy Forage Research Center, USDA-ARS, Madison, WI 53706

**Keywords:** Genomic Prediction, exome capture, population structure, winter survival, GBLUP, GenPred, Shared Data Resources

## Abstract

The lowland ecotype of switchgrass has generated considerable interest because of its higher biomass yield and late flowering characteristics compared to the upland ecotype. However, lowland ecotypes planted in northern latitudes exhibit very low winter survival. Implementation of genomic selection could potentially enhance switchgrass breeding for winter survival by reducing generation time while eliminating the dependence on weather. The objectives of this study were to assess the potential of genomic selection for winter survival in lowland switchgrass by combining multiple populations in the training set and applying the selected model in two independent testing datasets for validation. Marker data were generated using exome capture sequencing. Validation was conducted using (1) indirect indicators of winter adaptation based on geographic and climatic variables of accessions from different source locations and (2) winter survival estimates of the phenotype. The prediction accuracies were significantly higher when the training dataset comprising all populations was used in fivefold cross validation but its application was not useful in the independent validation dataset. Nevertheless, modeling for population heterogeneity improved the prediction accuracy to some extent but the genetic relationship between the training and validation populations was found to be more influential. The predicted winter survival of lowland switchgrass indicated latitudinal and longitudinal variability, with the northeast USA the region for most cold tolerant lowland populations. Our results suggested that GS could provide valuable opportunities for improving winter survival and accelerate the lowland switchgrass breeding programs toward the development of cold tolerant cultivars suitable for northern latitudes.

Switchgrass (*Panicum virgatum* L.) is a North American native perennial warm season grass and a promising cellulosic bioenergy feedstock. The United States Department of Energy (US DOE) recognized switchgrass as a model bioenergy feedstock owing to its higher biomass production potential, perennial growth habit and stress tolerance (Parrish and Fike 2005; Wright and Turhollow 2010). There are two ecotypes of switchgrass, lowland (tetraploid) and upland (tetraploid and octoploid) (Casler 2012; Lee *et al.* 2014; Parrish and Fike 2005). The lowland ecotype has generated considerable interest due to its higher biomass yield and late flowering characteristics relative to the upland ecotype (Casler *et al.* 2011; Wullschleger *et al.* 2010; Poudel *et al.* 2019).

Lowland cultivars may have double the biomass yield of upland cultivars when planted in southern locations (Casler 2012). As such, breeding programs in the northern USA are focused on adapting southern lowland germplasm for use in the northern USA, where winter conditions are considerably colder than in the southern USA. Two traits form the focus of these breeding programs: late flowering to extend the growing season and winter survivorship to ensure stand longevity. Three strategies are being employed to accomplish this goal: (1) the use of upland x lowland hybrids (Vogel *et al.* 2014; Poudel *et al.* 2019), (2) selection for late flowering within northern-adapted upland germplasm, and (3) selection for winter survivorship within late-flowering but unadapted southern lowland germplasm. The latter strategy is the focus of this research.

Lowland ecotypes planted in the northern latitudes exhibit very low winter survival. Lowland cultivars, when planted one hardiness zone north of their origin, resulted in 9 to 17% reduction in both biomass yield and survival (Casler *et al.* 2004). While winter survival (WS) can be improved by selectively saving surviving plants exposed to low-temperature stress, this approach requires several years for the completion of a single selection cycle and its success is highly dependent on the presence of winter conditions that generate the appropriate selection pressure. Moreover, selection based on controlled environments does not always reflect selection under realistic field conditions, due to differential gene expression (Dhanaraj *et al.* 2007). As such, the efficiency and effectiveness of controlled-environment selection may not represent the optimal solution. Genomic prediction and selection could potentially enhance switchgrass breeding for winter survival by reducing generation time (Lipka *et al.* 2014; Simeão Resende *et al.* 2014; Wong and Bernardo 2008) while eliminating the dependence on weather.

Genomic selection (GS) is emerging as a powerful tool in animal and plant breeding (Heffner *et al.* 2009). Through dense genome-wide marker coverage to predict the breeding value of complex traits (Meuwissen *et al.* 2001), it facilitates rapid selection of superior genotypes and accelerates breeding cycles. Several GS models such as genomic-best linear unbiased predictor (G-BLUP)(Hayes *et al.* 2009), ridge regression-BLUP (RR-BLUP) (Endelman 2011), reproducing kernel Hilbert space (RKHS) (Gianola and van Kaam 2008), Bayesian models (Park and Casella 2008) and random forest (RF) (Díaz-Uriarte and De Andres 2006) along with their regularization are being used to optimize gains in accuracy. Despite the role of prediction procedures, the efficiency of GS models is primarily affected by heritability of the trait, effective population size of the training set, the relationship between individuals in training and testing sets, and marker density (Calus *et al.* 2008; Habier *et al.* 2013).

Genomic studies relating predictive ability by combining multiple populations into a single training set are limited. Increasing the size of the training set by combining populations, generally improves prediction accuracies because the markers effects are estimated from a larger number of phenotypes, assuming some degree of homogenous population structure (Lorenzana and Bernardo 2009; VanRaden 2008). However, increasing size with unrelated individuals or with a heterogeneous population structure might lead to inconsistent linkage disequilibrium (LD) patterns between the marker and causal variants (Wientjes *et al.* 2013) and thus might reduce prediction accuracy. The use of high-density markers in GS predominantly captures small-effect quantitative trait loci (QTL) and outperforms the breeding efficiency based on phenotype or marker-assisted selection. Marker density is a critical component of GS predictive ability because a higher number of markers increases the probability that the causal variant will be in LD with at least some markers across the population (Calus *et al.* 2008; Goddard 2009). In a simulation study in cattle, combining populations had been beneficial in improving predictive ability at high marker density and was more accurate for traits with low heritability (de Roos *et al.* 2009).

The assessment of GS in switchgrass had previously been performed by Lipka *et al.* (2014), Ramstein *et al.* (2016), and Casler and Ramstein (2018) for morphological and biomass quality traits, which all demonstrated the possibility of increased gain per unit time. However, GS for winter survival has not been reported. In this study, we examined the potential of GS for winter survival in lowland switchgrass by combining multiple populations in the training set. Specifically, we assessed the performance of genomic prediction procedures and applied the selected model to two independent datasets for validation purpose in two contexts: (1) using indirect indicators of winter adaptation based on geographic and climatic variables of accessions from different source locations and (2) using winter survival estimates of the phenotype.

## Materials and Methods

### Training population

This study used metadata on 405 half-sib (HS) families from five separate lowland switchgrass experimental nurseries (Table 1) namely: Liberty (Ramstein *et al.* 2016), WS11L, WS08L (Evans *et al.* 2018a; Grabowski *et al.* 2017), WS09L, and Expresso. These five experiments represented multiple breeding populations of lowland or hybrid switchgrass. All nurseries were planted using a row-plot design with three or four replicates per location either in 2011 or 2012. Plots consist of one row of five plants on a 30 cm spacing with 90 cm between rows. The experiments were evaluated in a randomized complete block design (RCBD) at two locations: Arlington, WI (Plano silt loam; fine-silty, mixed, superactive mesic Typic Argiudoll) and Hancock, WI (Plainfield loamy sand; mixed, mesic Typic Udipsamment) or Mead, NE (Sharpsburg silt loam fine, smectitic, mesic Typic Argiudoll). Liberty was evaluated in Arlington, WI and Mead, NE; whereas WS08L, WS09L, and Expresso were evaluated in Arlington and Hancock, WI. The WS11L experimental nursery was the only exception, planted in a completely randomized design only at Arlington, WI. Plots were harvested every year after the first killing frost and biomass was removed at the time of harvest. Plots were fertilized with 100 kg N ha^-1^ in spring of each production year.

**Table 1:** Description of switchgrass source populations and half-sib families used for genomic prediction of winter survival (training data sets)

Population	Ecotype	Source of germplasm	Number of families
Liberty*^a^*	Lowland × Upland	Kanlow × Summer	111
WS11L	Lowland	Georgia and Alabama	36
WS08L*^b^*	Lowland	Eastern and Southern USA, Great Plains	130
WS09L	Lowland	North Carolina, South Carolina, Florida	69
Expresso	Lowland	Mississippi	59

a Ramstein *et al.* (2016).

b Evans *et al.* (2018a); Grabowski *et al.* (2017).

Individual plants were scored for survival approximately 7-10 days after the initial spring growth for two evaluation years: Spring 2014 and Spring 2015. Scoring was done by visual assessment of the percentage of living shoots on the scale of 0-20, where 0 dead, 1 = one green shoot, ..., and 20 = no winter damage (Supplemental Table 1). The best linear unbiased predictor (BLUP) of each half-sib family was estimated for each experiment considering all effects as random and using the following linear model:

$$y_{ijkl}=µ+l_{i}+f_{j}+r_{k\left(i\right)}+{\left(f\times r\right)}_{jk\left(i\right)}+{\left(l\times f\right)}_{ij}+{\left(f\times r\times l\right)}_{jk\left(i\right)}+\epsilon_{ijkl}$$

where: *y_ijkl_* is the response, *l_i_*, *f_j_* and *r_k(i)_* are the effects of location *i*, family *j* and block *k* nested within location *i* respectively, and *ϵ_ijkl_* are residuals. For WS11L, the following model was used:

$$y_{jkl}=µ+f_{j}+r_{k}+{\left(f\times r\right)}_{jk}+\epsilon_{jkl}$$

where: *y_jkl_* is the predicted response, *f_j_* and *r_k_* are the effect of family *j* and replication *k* respectively, and *ϵ_jkl_* are residuals. In order to account for experiment-wise phenotypic variation, the phenotypic value for genomic analysis was set to be non-centered means such that$\;y_{i}=\hat{\mu }+\hat{f_{j}}$.

### Genotype and Quality Control

The maternal parent of each HS family was genotyped using the exome capture platform. Exome capture sequence reads were cleaned using Cutadapt (https://cutadapt.readthedocs.org/en/stable/) and the FASTX toolkit (http://hannonlab.cshl.edu/fastx_toolkit/) and were aligned to the AP13 switchgrass reference genome (Version 1.1) using BWA-MEM (Li 2013). Variants were called using the GATK package (McKenna *et al.* 2010) and copy number variants were called using CNVnator (Abyzov *et al.* 2011). Genotype calling was implemented using the EM algorithm of Martin *et al.* (2010). Markers were filtered for the proportion of missing values lower than 20%; minor allele frequency greater than 1/2N, with N being the total number of individuals; and Hardy-Weinberg equilibrium (HWE) based on a chi-squared test p-value > 10^−4^. Missing observations after filtering, less than 5% of the total, were imputed by the population mean.

### Training (Calibration) Dataset

Of the 405 HS families, 368 HS families were used to develop GS models. Genotype data for 20 HS families were not available and 17 families were excluded from the analysis because genetically they were classified as the upland ecotype, as determined by structure analysis, or were classified as octoploid as demonstrated by the deviation in distribution of heterozygote biallelic SNP’s alternate allele frequencies from the mean value 0.5 to either 0.25 and/or 0.75.

The resulting matrix contained markers at 435,919 polymorphic sites for 368 HS families. The HS families in this dataset were collected from different breeding programs and are subject to strong population structure (Table 1, Supplemental Figure 1). A population structure based on Admixture (Alexander *et al.* 2009) was performed to identify potential population heterogeneity. Based on the results from the admixture analysis the training population was divided into three distinct population groups: Southern Lowland, Eastern Lowland, and Lowland x Upland, closely corresponding to the subdivisions shown earlier for the northern and southern association panels (Evans *et al.* 2018a).

Genomic heritability was calculated as the variance explained by the GBLUP model *i.e.*, $\frac{\sigma_{u}^{2}}{\sigma_{u}^{2}+\sigma_{e}^{2}}$, where $\sigma_{u}^{2}$ is the variance of random effects of markers and $\sigma_{e}^{2}\;$is the residual variance.

Each panel was evaluated for prediction accuracy using five GS models. The standard statistical model for genomic prediction was genomic BLUP (GBLUP). The GBLUP model is defined as

y=μ+Zu+e

where y is the HS family BLUP, µ is the grand mean, Z is the marker identity matrix; u ∼ N(0, Kσ^2^_u_), K being the n x n genome estimated relationship matrix and e is the residual. The GBLUP model assumed additive, linear and homoscedastic effects of the markers and was fitted using rrBLUP (Endelman 2011) R package. In addition, Bayesian models BayesA (BA), BayesB (BB) and Bayesian lasso (BL) were considered (Meuwissen *et al.* 2001; Park and Casella 2008). The Bayesian models were fitted using BGLR R package (Pérez and de los Campos 2014) with 20000 iterations and 5000 burn-in. To account for heteroscedastic and nonlinearity, a machine learning method, Random Forest (RF), was implemented. The RF model was fitted with 200 decision trees and 1/6 of total markers as the number of variables to be randomly sampled as candidates at each split using R package randomForest (Díaz-Uriarte and De Andres 2006).

The prediction accuracy in the training set was determined as the Pearson coefficient of correlation between the phenotypic BLUP and predicted GEBV as determined using a fivefold CV scheme (Legarra *et al.* 2008). In this scheme, the observations were randomly divided into five non-overlapping groups, four of which were used as a pseudo-training set and the remaining group was assigned as a testing set. The CV was replicated 20 times and the average was used as the predicted GEBV for GBLUP model and un-replicated for all other models. The GEBV generated from heteroscedastic and/or nonlinear models were compared to those from the GBLUP model using Dunnett’s paired *t*-test using R package multcomp (Hothorn *et al.* 2017).

### Validation Datasets

Several training models were evaluated for validation performance using two independent datasets: validation datasets #1 (VDS #1) and #2 (VDS #2) (Tables 2 and 3). All validations were conducted using three different training sets: All Populations (Southern Lowland, Eastern Lowland, and Lowland x Upland), All Lowland (Southern Lowland and Eastern Lowland), and Southern Lowland populations.

**Table 2:** Mean winter survival percentage and family-mean broad sense heritability (H^2^) for five switchgrass populations and five population groups, based on field data from Spring 2014 or 2015 at Arlington and Hancock, WI

	Spring 2014		Spring 2015	
	Mean winter Survival	H^2^*^a^*	Mean winter Survival	H^2^
	%		%	
Liberty (111)*^b^*	36.7 ± 0.7	0.24	49.3 ± 0.3	0.13
WS11L (36)	19.8 ± 1.1	NA	29.4 ± 0.9	NA
WS08L (130)	37.4 ± 0.9	0.72	23.1 ± 0.7	0.71
WS09L (69)	5.9 ± 0.5	0.62	3.1 ± 0.4	0.68
Expresso (59)	0.5 ± 0.1	0.87	0.2 ± 0.1	0.92
All populations (368)	24.8 ± 1.1	0.95	24.5 ± 1.1	0.93
All Lowland (257)	19.6 ± 1.4	0.99	13.8 ± 1.0	0.89
Southern Lowland (168)	5.3 ± 0.8	0.98	5.6 ± 0.8	0.85
Eastern Lowland (89)	46.5 ± 1.5	0.63	29.3 ± 1.2	0.61

a NA = No heritability estimates due to evaluation of this population at only one location.

b The number inside the parenthesis represents number of half-sib families.

**Table 3:** Mean GBLUP prediction accuracies for five combinations of switchgrass populations based on winter survival in either spring 2014 or 2015

Population name	Number of families	Number of SNP markers	Spring 2014	Spring 2015
All Populations	368	434,919	0.88	0.91
All Lowland	257	400,699	0.87	0.77
Southern Lowland	168	204,636	0.17	0.43
Eastern Lowland	89	239,612	0.15	0.23
Lowland x Upland	111	95,397	0.20	0.02

Validation dataset #1 consisted of 253 individuals comprising 43 populations or accessions, which were defined as lowland based on structure analysis and were independent of the training set (Evans *et al.* 2018a; Grabowski *et al.* 2017). Accuracy of prediction was calculated as a Pearson’s coefficient of correlation between adjusted genome estimated breeding values (GEBV) and climatic or geographic variables: 30-yr mean minimum temperature of the coldest month (http://www.worldclim.org/), latitude, and longitude of the site of origin of the source population (Supplemental Table 2). Adjusted GEBV was calculated by fitting a linear model to account for the effect of individuals within the population.

This dataset is subject to strong population structure. Therefore, additionally, a mixed population matérn (MPM) model (Ramstein and Casler 2017) was performed to account for population heterogeneity using the following linear model

$$y=\mu +u+e,u∼N\left(0,(\Omega n○ZZ^{\prime }\;\sigma_{u}^{2}\right)\;\text{and}\;e∼N\left(0,\sigma_{e}^{2}\right),\Omega n=K_{v,h}(P_{i},P_{j})$$

where: *y* is the n-vector of phenotype, *u = Zµ*, *Z* is marker matrix (n x m), ○ is the element-wise product, Ω*n* is n x n covariance matrix depicting population differentiation among individuals, *K* is the matérn kernel function with *v* and *h* as scale and shape parameters and (*P_i_*, *P_j_)* marker estimated principal components (pc) for any pair of individuals (*i,j*). This model is denoted by GBLUP-MPM throughout this paper.

GBLUP-MPM model is the extension of the GBLUP model which accounts for population heterogeneity using principal components calculated from marker data on the combined (calibration and validation) dataset. The number of principal components (d) to be used was determined based on the variance explained by each component and number of clusters revealed through the principal component plot. The optimization of *v* and *h* parameters in *Ωn* were estimated by maximizing the restricted likelihood of the above model using R function constrOptim, given the starting point for (*v_o_*, *h_0_*) as (0.5, 0.5). The Euclidean distance matrix (n x n covariance matrix) depicting population differentiation among individuals was estimated from *d*, *v*, and *h* using the matérn function in R.

Validation dataset #2 was a subset of data, mostly consisting of natural populations originated from Southern USA (Supplemental Table 3) from a winter survival study in Wisconsin (unpublished data). Seedlings that were 16 weeks of age were transplanted in June 2015 at two locations: Arlington, WI and Madison, WI (Kegonsa silt loam; fine-silty over sandy or sandy-skeletal, mixed, superactive, mesic Mollic Hapludalf), in a randomized complete block design with eight blocks and 25 plants per plot. Plants were spaced 0.3 m apart within rows and rows were 0.9 m apart. The winter survival of individual plants was scored in Spring 2017, as described for the training set. A random set of 178 individuals representing 23 populations were genotyped as described for the training set (Supplemental Table 3).

The winter survival phenotypic values for populations were estimated using the following random effect model.

$$y_{ijk}=p_{i}+b_{j}+{\left(p\;\text{x}\;b\right)}_{ij}+\epsilon_{ijk}$$

where: *y_ijk_* is the predicted response, *p_i_* and *b_j_* are the effect of population *i* and block *j* respectively, and *ϵ_ijk_* are residuals. The effect of location and location x population were significant (α < 0.05) for winter survival, so locations were analyzed separately for prediction purposes.

Two prediction procedures were used for this validation set. First, phenotypic winter survival scores for the exact same individuals genotyped in the VDS #2 (N = 178) were used to calculate prediction accuracy. Second, adjusted GEBV for populations were computed on a population basis independently for the Arlington and Madison locations. Adjusted GEBV and phenotypic BLUP values were then used to calculate prediction accuracies on a population basis for each location.

### Data availability

Genotype and phenotype data of training set and validation dataset #2 and supplemental materials necessary for confirming the conclusions presented in the article are available at Figshare. Geno_TS.rds is the training dataset consisting of 368 individuals at 434,254 markers, geno_vds2.rds is the validation dataset #2 with 178 individuals at 460,822 markers position. Pheno_TS.csv and pheno_VDS2.csv are the corresponding phenotype data files for the training set and the validation data set #2.

The genotype data used for validation dataset #1 can be accessed through Dryad Digital Repository under the name: snipe_slap_sapper_filtered_biallelic_snps_final_reheader.txt.bz2 (Evans *et al.* 2018b) (https://doi.org/10.5061/dryad.mp6cp). Supplemental material available at Figshare: https://doi.org/10.25387/g3.7336187.

## Results

### Phenotypic variability for winter survival

There was considerable variation in winter survival among populations, ranging from <1% in Expresso to almost 50% in Liberty, a population derived from lowland x upland hybrids (Table 2). As expected, HS families from Liberty had the highest mean winter survival in both years, likely due to the upland genome in their ancestry. Liberty had the lowest broad-sense heritability compared to the other populations, all of which had broad-sense heritability >0.62. The lowland populations derived from southern USA germplasm possessed considerable phenotypic variability for winter survival, both among and within populations, the latter evidenced by the relatively high heritability values.

The structure analysis of these genotypes, using the DNA marker matrix, resulted in a strong split of lowland individuals between an Eastern Lowland population and a Southern Lowland population (Supplemental Figure 1), which was similar to results of Evans *et al.* (2018a). The Eastern Lowland population derives largely from New York and New Jersey (Evans *et al.* 2018a), while the Southern Lowland population derives from the Carolinas, the Gulf Coast, and the southern Great Plains. Again, largely as expected, the Eastern Lowland population had a higher mean winter survival compared with the Southern Lowland population (Table 2).

### Genomic structure and relatedness

**Figure 1:**
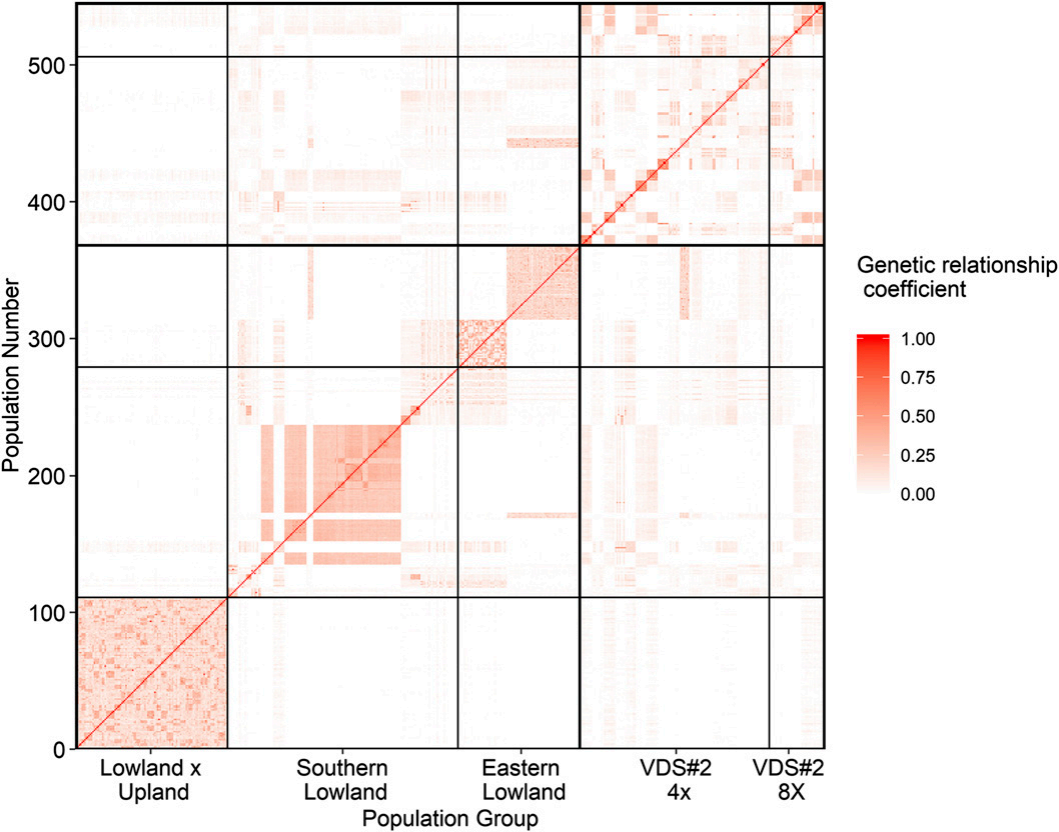
Marker-derived genomic relationships (identify-by-state, IBS) for 368 switchgrass half-sib families in the genomic prediction training set (bold bordered area) and Validation data set #2 (VDS #2). The label “Population number” on the y-axis refers to an arbitrary number assigned to 368 half-sib families of the training set plus 178 individuals in VDS #2.

The distribution of minor allele frequency (MAF) of the selected markers in the Eastern Lowland and Southern Lowland populations differed from that of the Lowland x Upland population. The 95^th^ percentile of the markers in Lowland x Upland had MAF < 0.39 while it was only 0.18 and 0.29 in Eastern Lowland and Southern Lowland, respectively (Supplemental Figure 2) indicating the proportion of alleles at or near fixation was higher in natural populations than the bi-parental hybrid population. Similarly, the pattern of LD decay in the Lowland x Upland population was slower than the other populations (Supplemental Figure 3) in accordance with the smaller effective population size of the Lowland x Upland population. The within-population identity-by-state (IBS) was highest in Eastern Lowland (0.35) followed by Lowland x Upland (0.17) and lowest in Southern Lowland (0.07). The among-population coefficients of IBS were nearly zero, which can be inferred from Figure 1.

### Assessment of genomic prediction procedures

The assessment of genomic models was conducted separately for five population groups: All Populations, All Lowland, Southern Lowland, Eastern Lowland, and Lowland x Upland. Prediction accuracies for the GBLUP model were highly variable across population groups, ranging from 0.02 to 0.91 (Table 3). Prediction accuracies were highest for the most diverse population groups with the greatest population size. Prediction accuracies were lowest for the Lowland x Upland population, probably due to the low heritability of winter survival in this population. None of the other genomic prediction models resulted in any improvement over the GBLUP model (data not shown).

### Validation of genomic prediction models (VDS #1)

Validation data set #1 was composed of exclusively lowland populations, most of which were of southern origin (Supplemental Table 2). Therefore, it was of interest to determine which of three potential training sets provided the best predictions of GEBV from minimum temperature, latitude, or longitude of the site of origin for the populations in Supplemental Table 2. Correlation coefficients (r) of GEBV with minimum temperature and longitude were always negative and those with latitude were always positive, with the only exceptions occurring for the Southern Lowland population (Table 4). Predictions using GBLUP-MPM model were more accurate than GBLUP model as evidenced with increased |r| value by up to 0.19 while using models based on All Populations and the All Lowland population. The underrepresentation of Eastern-origin individuals in the Southern Lowland training set probably led to the inconsistency in prediction accuracies for that population.

**Table 4:** Correlation coefficients between genomic estimated breeding values (GEBV) for winter survival (Spring 2014 or 2015) and climatic or geographic variables of switchgrass accessions comprising Validation data set #1

		Min Temp*^a^*		Latitude		Longitude	
Training set	Method	Spring 2014	Spring 2015	Spring 2014	Spring 2015	Spring 2014	Spring 2015
All Populations	GBLUP	−0.23*^b^*	−0.04	0.57	0.32	−0.76	−0.60
All Lowland	GBLUP	−0.27	−0.15	0.61	0.47	−0.78	−0.70
Southern Lowland	GBLUP	−0.26	−0.17	0.09	−0.05	0.25	−0.19
All Populations	GBLUP-MPM	−0.37	−0.10	0.71	0.43	−0.80	−0.69
All Lowland	GBLUP-MPM	−0.38	−0.29	0.72	0.62	−0.80	−0.74
Southern Lowland	GBLUP-MPM	−0.26	−0.17	−0.05	0.07	0.25	−0.19

a Min Temp = 30-yr mean minimum temperature of the coldest month at the site of origin of source population.

b Critical values for *P* < 0.01 significance are: 0.14 for All Populations, 0.17 for All Lowland, and 0.20 for Southern Lowland.

**Figure 2:**
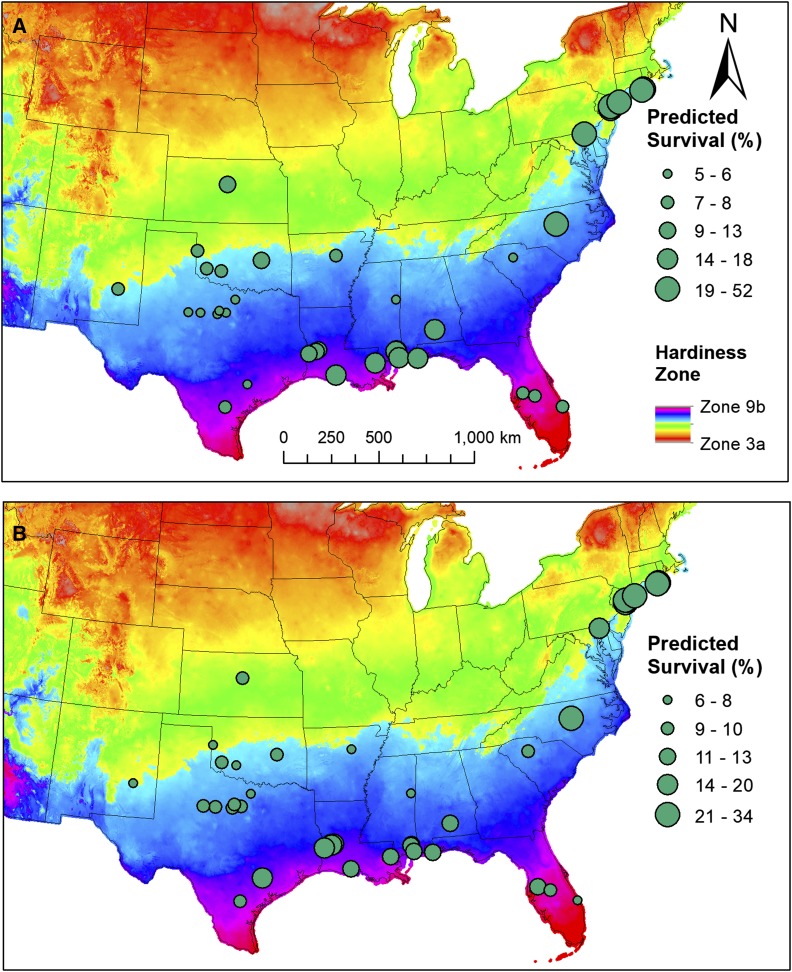
Adjusted genomic estimated breeding values (GEBV) of the populations in Validation data set #1 (VDS #1) plotted over the map of USDA hardiness zones. A. Predicted winter survival 2014 and B. Predicted winter survival 2015.

The highest and most consistent correlation coefficients across all variables, years, and methods were observed for the All Lowland population as the training set (Table 4). Multiple linear regressions of the GEBV obtained using this model with geographic variables latitude and longitude were performed to observe the relative contribution of each variable on winter survival. The minimum temperature was not included in the model because of collinearity (*r* = 0.88) with latitude. Longitude was statistically significant in all combination of years and methods (α < 0.01) however latitude was not significant for the GBLUP method in 2015 (α > 0.05, Table 5). However, the prediction method GBLUP-MPM improved the prediction such that both geographic variables were statistically significant (α < 0.01) in both years. By accounting for population heterogeneity, the fitness of these models, as explained by adjusted R squared, increased by 0.11 and 0.14% for the 2014 and 2015 datasets, respectively. The map representing the origin of the populations with their adjusted GEBV using GBLUP-MPM method is presented in Figure 2. This result indicated that winter survival of lowland switchgrass populations had a linear relationship to the origin of the population *i.e.*, gradually increasing from south to north and from west to east.

**Table 5:** Coefficients of multiple linear regression for regressions of adjusted genomic estimated breeding values (GEBV) predicted from latitude and longitude of the origin for all lowland switchgrass populations within Validation data set #1

		Spring 2014			Spring 2015		
	Source of variation	Estimate	t-value	P-value	Estimate	t-value	P-value
GBLUP	Intercept	67.14	3.05	**	48.64	3.12	**
	Latitude	1.13	3.15	**	0.37	1.45	NS
	Longitude	0.98	6.47	***	0.52	4.86	***
	R^2^	0.68			0.48		
GBLUP-MPM	Intercept	46.88	2.51	*	32.23	2.53	*
	Latitude	1.67	5.48	***	0.67	3.23	**
	Longitude	0.98	7.57	***	0.47	5.28	***
	R^2^	0.79			0.62		

* , **, *** Significantly different from zero at the 0.05. 0.01, or 0.001 probability level, respectively. NS = Non-significant.

### Validation of genomic prediction models (VDS #2)

The predictive ability of models developed using training sets All Populations or All Lowland, when applied to all individuals of VDS #2 using the GBLUP individual prediction method, was less than 0.20 (Table 6). The low accuracies for these validations were most likely affected by two factors: inclusion of unrelated individuals in the training set (the VDS #2 mostly had a southern origin) and possible inclusion of octoploids in the validation set (see discussion). The use of exclusive southern-origin populations in the training set (Southern Lowland) significantly increased the predictive ability of the genomic prediction model for all prediction methods. This increase in the prediction accuracy could be supported by the fact that the populations used in VDS #2 were mostly natural populations collected from the southern USA and had a closer resemblance to the individuals in the Southern Lowland population (Figure 1).

**Table 6:** Prediction accuracy for winter survival of switchgrass populations in Validation data set #2. Predictions were based on GBLUP methods applied to training data sets from Spring 2014 or 2015 and applied to independent populations evaluated in 2017 at Arlington or Madison, WI

				All individuals		Putative tetraploids only	
Training set	GBLUP prediction method*^a^*	Number of families	Number of SNP markers	Spring 2014	Spring 2015	Spring 2014	Spring 2015
All Populations	Individual	368	200,933	0.05	0.19	0.17	0.01
	Arlington			0.34	0.14	0.46	0.17
	Madison			0.05	0.14	0.15	0.00
All Lowland	Individual	257	176,581	0.01	0.13	0.20	0.01
	Arlington			0.34	0.14	0.46	0.17
	Madison			0.05	0.14	0.15	0.00
Southern Lowland	Individual	168	141,344	0.51	0.19	0.51	0.25
	Arlington			0.79	0.46	0.84	0.49
	Madison			0.65	0.24	0.64	0.28

a Individual: Prediction of individuals that were exactly sequenced. 178 Individuals representing 23 populations from location Madison were sequenced and All individuals comprised all 178 individuals and putative tetraploids comprised 138 individuals. Arlington: prediction of population GEBV at the Arlington location (23 populations for All individuals and 18 populations for putative tetraploids only); Madison: prediction of population GEBV at the Madison location (23 populations for All individuals and 18 populations for putative tetraploids only). GEBV = genomic estimated breeding value.

For all combinations of the training set and GBLUP prediction methods, the prediction accuracies for the dataset consisting only putative tetraploids were higher than for the dataset consisting of all individuals for 2014 data, but not for 2015 data. Prediction accuracies based on Arlington data were always higher than for Madison data which is probably associated with stronger winter pressure at Arlington due to colder temperatures. Arlington was also one of the sites used for calibrating the training model.

## Discussion

The winter of 2013/14 had consistently lower minimum temperatures throughout the winter than the 30-year normal minimum temperature (data not shown, https://www.ncdc.noaa.gov/cdo-web/), suggesting that relatively high selection pressures were likely exerted on these nurseries in the training set. This was evidenced by relatively high phenotypic variability between and within populations (Table 2). The winter survivorship scores were highly correlated between evaluation years 2014 and 2015 (85%), as the effects from the second winter were largely additive to the evaluation from the first year. However, the data were analyzed separately for each year as a test of consistency in prediction and due to a significant genotype x year interaction.

This study used meta-data from five experimental nurseries comprising multiple populations tested in multiple environments. Several studies have proposed that multiple-environment GS models can capture the variability due to the environment and increase the performance of genomic prediction (Burgueño *et al.* 2012; Cuevas *et al.* 2016; Lado *et al.* 2016). The common strategies to deal with genotype x environment interaction (GxE) are to ignore, reduce or exploit it (DeLacy *et al.* 1996; Eisemann *et al.* 1990). For each experiment in this study (except WS11L, which was evaluated in only one location), there was a significant GxE interaction. The strategy we employed here was to reduce the GxE interaction by modeling it during the estimation of phenotype (Lado *et al.* 2016). We did not model to exploit the GxE in this study because the goal of this study was to determine the possibility of GS for winter survival and to use genomic prediction of population performance to develop more broadly adapted switchgrass populations across multiple locations (Lal 2013). Candidate switchgrass cultivars to be developed from this research will require fairly broad adaptation across hardiness zones 4-6 before they can be released for commercialization.

### GS prediction procedures and validation

Most of the GS studies consider optimization of the genomic prediction procedures to improve the performance of prediction (Moser *et al.* 2009). There are several reported studies which used marker data transformation for GS. While marker data transformation by accounting for LD and weighing marker effects may be useful in reducing the effective number of markers (Ramstein *et al.* 2016), it did not significantly or consistently improve the prediction accuracy for dry matter yield in switchgrass. Similarly, higher marker density had an important role on the reliability of predictive ability while predicting across multiple populations because higher numbers of markers will increase the probability that the causal variant will be in LD with at least some markers across the populations (Calus *et al.* 2008; Goddard 2009). In many studies, combining different groups of related populations as part of the training set has shown to increase prediction accuracy (Schulz-Streeck *et al.* 2012; Technow *et al.* 2013; Zhao *et al.* 2012). Therefore, we incorporated all markers in our model regardless of marker effects and LD (de los Campos *et al.* 2013; Ramstein *et al.* 2016). The higher predictive ability of GBLUP over other models in this study could be because of the low LD (Supplemental Figure 3) among markers, supporting the results by Zhong *et al.* (2009) who reported GBLUP models to perform better than Bayesian models when markers were in weak LD and the individuals were highly related.

The model based on a training set that includes the Lowland x Upland population had lower predictive ability compared to the model based on the exclusively Lowland-ecotype training set when evaluated against the two independent-validation datasets (VDS #1 and VDS #2). This is likely due to the strong genetic dissimilarity of the Lowland x Upland population compared to the other populations (Figure 1), owing to the differences in divergence between natural lowland populations and the selectively bred population that includes a significant dose of upland germplasm in its parentage (Zhang *et al.* 2011a). These results are similar to those of Ramstein *et al.* (2016), who demonstrated low prediction accuracies between a diverse upland population and the Lowland x Upland population used in our study. Such low prediction accuracies might be due to the low degree of IBD between ecotypes (Würschum *et al.* 2013) (Figure 1) and differences in mean performance for the trait between training and validation sets (Windhausen *et al.* 2012).

Switchgrass ecotypes, cultivars, and breeding populations are highly sensitive to GxE interactions. Latitude and photoperiod combine to form the most important factor in determining adaptation. In general, ecotypes or cultivars planted more than one hardiness zone north or south of their origin are frequently subject to significant reductions in both biomass yield and survival (Casler *et al.* 2004; Casler *et al.* 2007; Hopkins *et al.* 1995). Breeding for broad adaptation, including selection for winter survivorship under optimal conditions (*i.e.*, with favorable selection pressure) can alter this relationship by broadening the adaptation of some populations (Casler *et al.* 2018). Longitude is also a significant factor influencing GxE interactions of switchgrass, largely in the form of a moisture gradient from the Great Plains to the Atlantic Seaboard (Casler *et al.* 2004; Casler *et al.* 2019). The prediction model based on all lowland populations was highly effective at predicting GEBV for winter survival in Wisconsin, based solely on latitude and longitude of the site-of-origin (Tables 4 and 5). These results indicate that genomic prediction methods can be utilized to target germplasm exploration efforts within regions that possess the greatest potential for contributing favorable alleles to the breeding program. Previous germplasm exploration efforts have been focused vaguely and broadly on collecting lowland ecotypes from across the entire southern USA to capture the late flowering trait (Casler 2012).

Using the validation results for VDS #1, future efforts can be directed to more specific sites and regions with a higher probability of containing alleles useful for improving freezing tolerance of northern-adapted lowland populations, as illustrated in Figure 2. Indeed, inspection of Figure 2 suggests that genomic prediction may not be a necessary component for targeting switchgrass exploration and collection to broaden the allelic base for winter hardy lowland germplasm. Within the Great Plains, hardiness zone is the key component, with the most hardy germplasm coming from the most northern hardiness zones. Likewise, there is a clear trend within hardiness zones 8 and 9, that the most winter hardy germplasm tends to be more eastern in origin. Alleles for winter survivorship and genotypes capable of surviving Wisconsin winters appear to reside within nearly all investigated populations, at varying frequencies, but the more northern and eastern the origin, the higher the frequency of these alleles and genotypes.

An additional application of these results will be in the utilization of genomic prediction procedures within the breeding program, *i.e.*, accelerating the breeding of freezing-tolerant lowland populations through genomic selection. For this application, the Southern Lowland prediction models appear to have the most applicability, as evidenced by the highest prediction accuracies likely due to the high mortality rates associated with populations of southern origin (Table 2). The Eastern Lowland populations were not useful in this regard, due to their relatively low mortality in the field studies, owing to their northern and eastern origin (Table 2, Figure 2). Furthermore, these Eastern Lowland populations tend to be early to moderate in flowering date, as determined by their evolution at 40 to 42°N latitude.

Previous studies of GxE interactions in switchgrass, demonstrating strong adaptational responses associated with both latitude and longitude, have been largely based on upland cultivars and ecotypes (Casler *et al.* 2007; Hopkins *et al.* 1995). This study expands upon those results, demonstrating that these adaptational responses also apply equally importantly to genotypes within the lowland ecotype of switchgrass. Furthermore, Figure 2 also demonstrates that most lowland accessions of switchgrass possess some alleles for winter survivorship in hardiness zones 4 and 5, as well as a small frequency of genotypes capable of surviving winters in these hardiness zones. Remnant genetic variability for winter survivorship within Southern Lowland populations is likely due to the relatively short period of time since the last glacial maximum when northern and southern populations, as well as upland and lowland ecotypes, were compressed together into a narrow adaptational zone (Zhang *et al.* 2011b). Hybridization between extreme genotypes was frequent during glacial maxima, essentially creating wide hybrids and new genotypes that were useful in repopulating prairie and savanna ecosystems during the past 11,000 years. The polyploid nature of switchgrass is likely an important factor, at least partly responsible for the remnant variability for extreme winter survivorship within these Southern Lowland populations.

### Effect of the octoploid and tetraploid admixture in prediction accuracies

Flow cytometry of 11 populations within VDS #2 revealed an admixture of tetraploids and octoploids, with two out of eleven populations classified as octoploids. Genomic analysis was performed to determine the ploidy of all populations similar to that by Lu *et al.* (2013), using allelic read depth counts to generate apparent segregation ratios. For each individual, the reference allele frequency at each marker position was calculated as a ratio of reference allele read depth to the total read depth. A distribution of the reference allele frequency of heterozygous markers (segregating in a 1:2:1 Mendelian ratio) was plotted. All tetraploids had a peak at allele frequency 0.5. Conversely, the individuals which were octoploid had a peak at allele frequency 0.75 (Supplemental Figure 4) (Evans *et al.* 2018a; Grabowski *et al.* 2017). The determination of ploidy on the remaining individuals was based on similarity of segregation ratios to known tetraploid types or octoploid types. Based on these observations, 40 individuals from five populations were predicted to be octoploid.

Because there are very few reported lowland switchgrass that are octoploids (Zalapa *et al.* 2011; Zhang *et al.* 2011a), population structure analysis was required to identify the origin of these five populations. A combined population structure analysis of VDS #2 with the northern association panel (Evans *et al.* 2018a) revealed a hybrid origin of these five populations (Supplemental figure 1B), similar to observations made by Zhang *et al.* (2011a). The five VDS #2 octoploids populations in this study originated from Alabama, Georgia, Mississippi and Tennessee, where most of the intermediate octoploids accessions originated, and where both upland and lowland genotypes are known to be adapted (Zhang *et al.* 2011a).

The octoploid populations in this study had higher average predicted winter survivorship than tetraploids when the training set containing all lowland populations was used. This is likely due to their hybrid origin, containing significant upland genomic content, and unlikely due to ploidy *per se*. This result was in contrast to the phenotypic value recorded from the field evaluation and likely had a small adverse impact on predictive accuracy. Higher ploidy levels are generally associated with increased photosynthetic activity and greater stress tolerance in several crops (Joseph *et al.* 1981; Warner *et al.* 1987), while some reports indicated a decrease in photosynthesis activity (Austin *et al.* 1982). However, in switchgrass, higher ploidy (hexaploid) was reported to have lower photosynthetic activity than tetraploids (Wullschleger *et al.* 1996). Irrespective of the photosynthetic activity, the field evaluation of populations in VDS #2 demonstrated no differences in winter survivorship between octoploid and tetraploid populations that originated from the Southern USA. Due to the small differences in prediction accuracy between the full validation set and the tetraploid-only validation set (Table 6), it appears that these genomic prediction equations are relatively insensitive to ploidy. Rather, the geographic source of populations, reflecting their underlying genetic makeup, diversity, and linkage relationships, are the more important determinants of prediction accuracies within independent validation sets.

The overall aim of this study was to assess the GS models for predicting winter survivorship in lowland switchgrass populations. The measurement of winter survival under field conditions is very challenging in switchgrass because it is highly dependent on the presence of winter conditions that generate appropriate selection pressure. This was clearly evident in the evaluation of the VDS #2 population, with experienced <5% mortality following the winter of 2015/2016, compared to 50–75% following the winter of 2016/2017, even though the average minimum temperature during these two winters was similar. This limitation could be addressed to some extent by conducting selection in a controlled environment, but controlled environments do not often mimic real field conditions. Field grown plants are exposed to a varying light spectrum, light intensities, wind pressure, variable snow cover, and biotic stresses that are not present in controlled environments, resulting in differential gene expression (Dhanaraj *et al.* 2007; Gusta and Wisniewski 2013). Furthermore, the performance of perennial grass species cannot be effectively evaluated in a single growing season, as evidenced by multiple short-term breeding cycles leading to relaxed selection pressure for winter survivorship in high-digestibility switchgrass populations (Vogel *et al.* 2002). In general switchgrass breeding requires 3 to 7 years for the completion of a single phenotypic selection cycle (Casler 2012) and field evaluations should occur over a minimum of three winters (Casler *et al.* 2019).

Prediction accuracies, based on validations with independent populations in this study showed a high degree of predictive ability, within the range for which GS is thought to be beneficial for improving the efficiency of breeding programs (Simeão Resende *et al.* 2014). Results from the VDS #1 population indicate that it is possible to target sites for additional lowland switchgrass germplasm collection in the northern Great Plains, certain regions along the Gulf Coast, and in the northeastern USA. Similarly, the validation results from the VDS #2 population indicated that GS could be confidently used for predicting winter survivorship of individual genotypes within breeding populations, serving to both (1) accelerate the breeding program and (2) provide a basis for consistent selection pressure that does not necessarily depend on waiting for optimal selection conditions in the field.
